# *In situ* muscle pre-activation shifts the lateral gastrocnemius muscle–tendon unit to rely on active fascicle lengthening to absorb peak power input

**DOI:** 10.1242/jeb.251324

**Published:** 2026-03-10

**Authors:** Daisey Vega, Christopher J. Arellano

**Affiliations:** ^1^ Department of Orthopaedic Surgery, University of Arizona, Tucson, AZ 85724-5064, USA; ^2^ Department of Biomedical Engineering, University of Arizona, Tucson, AZ 85724-5064, USA

**Keywords:** Force, Elastic elements, Activation timing, Strain, Stretch, Mechanical energy

## Abstract

Muscle–tendon units (MTUs) tend to exploit their elastic elements to meet a range of energy-absorption and power input demands, but the extent of this may depend on how the muscle produces force. Muscle pre-activation is a habitual strategy observed *in vivo* during energy-absorbing demands, but it remains a question whether pre-activation alters the power input demands among elastic elements and muscle fascicles. To determine the effect of pre-activation on peak power input demands, we conducted *in situ* experiments using sonomicrometry and a linear actuator to simulate a pre-activation strategy in the lateral gastrocnemius MTU of wild turkeys (*n*=6). Onset timing of muscle activation was manipulated to start (1) simultaneously with or (2) before an active MTU stretch (i.e. no pre-activation versus with pre-activation). During MTU stretch, we quantified a peak power input decoupling ratio to determine the relative power input between muscle fascicles and elastic elements. We found that muscle pre-activation decreased the decoupling ratio (mean±s.d., 0.68±0.09 versus 0.56±0.11; *P*=0.015; Cohen's *d*=1.49), signifying that muscle fascicles absorbed a greater percentage of total MTU peak power input. We also found that the MTU generated greater force with pre-activation by relying more on active fascicle lengthening during the late phase of MTU stretch, which allowed for greater peak power input capacity of the MTU. These findings highlight how a simple shift in muscle activation timing can prime the MTU to deal with greater peak power input during energy-absorbing activities.

## Teaser

**Summary:** A muscle pre-activation strategy during *in situ* stretch experiments reveals how a muscle–tendon unit deals with high rates of energy-absorbing demands.

## INTRODUCTION

Muscle pre-activation is a neuromuscular strategy observed *in vivo* across multiple species during tasks requiring mechanical energy absorption, such as running, landing and even ball catching (Lacquaniti and Maioli, 1989; Santello and McDonagh, 1998; Santello et al., 2001; Azizi and Abbott, 2013; Konow and Roberts, 2024; Müller et al., 2010; Borrelli et al., 2020; Funase et al., 2001). In the case of a landing task, a high mechanical demand is placed on the lower limbs at impact and increases with greater heights as a result of a greater conversion of potential to kinetic energy as the body's center of mass (COM) accelerates downward. During impact, the muscle–tendon units (MTUs) of the lower limbs are faced with the challenge of absorbing and dissipating mechanical energy, which can vary in magnitude and rate (Devita and Skelly, 1992; Zhang et al., 2000; McNitt-Gray, 1993). A significant contributor to energy absorption is the ankle MTUs (e.g. gastrocnemius) (Devita and Skelly, 1992; McNitt-Gray, 1993; Zhang et al., 2000), which can be attributed to the elastic elements' ability to rapidly absorb energy (Konow et al., 2012; Konow and Roberts, 2015). However, to effectively absorb varying rates of energy under various conditions, there is likely a complex interaction that arises between the elastic elements and muscle fascicles. Studies have shown that when humans land from heights that range from 0.2 to 1.0 m (∼1 times their COM height), they activate their lower limb muscles before impact, and this pre-activation strategy is attributed to achieving increased muscle, limb and joint stiffness (Dyhre-Poulsen et al., 1991; Santello and McDonagh, 1998; Santello et al., 2001; Santello, 2005). Similarly, when turkeys are tasked with extreme landing heights of 1.5 m (∼3 times their COM height), they also increase their muscle pre-activation duration, which coincides with increases in leg stiffness and peak muscle force prior to ground impact (Konow and Roberts, 2024). Toads also use a muscle pre-activation strategy to shorten their muscle fascicles prior to landing, which accommodates greater lengthening strains associated with increased jumping distances (Azizi and Abbott, 2013). Overall, *in vivo* studies reveal that muscle pre-activation can modulate the amount of force and shortening prior to the need to absorb energy, but it's not clear how this strategy may alter the contribution of the elastic elements and active muscle fascicles to deal with a rapid rate of energy absorption demand during an active stretch.

*In situ* experiments have revealed that during active MTU lengthening, elastic elements can undergo significant stretch, which allows them to decouple muscle fascicle length changes from MTU length changes (Griffiths, 1991; Roberts and Azizi, 2010). Experiments by Griffiths (1991) show that during slow to moderate stretches (i.e. 20–40 mm s^−1^) of the MTU, muscle fascicles produce force while shortening, revealing that elastic elements take up the initial stretch imposed on the MTU (Griffiths, 1991). However, if muscle fascicles remain activated and the duration of the stretch continues, muscle fascicles will eventually transition to lengthening for the remainder of the stretch. When analyzing the decoupling of length changes from an energy-based framework, Roberts and Azizi (2010) reveal that muscle fascicles absorb less peak power input compared with the total peak power input of the MTU (Roberts and Azizi, 2010). This discrepancy is owed to the elastic elements' ability to rapidly absorb and store elastic energy, which lowers the rate at which muscle fascicles actively lengthen and dissipate energy. Yet, faster stretches (e.g. 100 mm s^−1^) of the MTU and longer durations of the activated muscle fascicles during the stretch (e.g. 50 versus 500 ms) predispose muscle fascicles to produce force while lengthening (Griffiths, 1991; Roberts and Azizi, 2010), which shifts the reliance of energy absorption and dissipation to the muscle fascicles. Therefore, this would suggest that the extent of peak power inputs to the muscle fascicles and elastic elements can be modulated by parameters of stretch and activation. Overall, the insights derived from classic *in situ* lengthening experiments have significantly contributed to our understanding of how elastic elements can play a critical role in an MTU's ability to deal with energy-absorbing demands. However, an overlooked feature of these experiments has been the simultaneous timing between the start of MTU stretch and the onset timing of muscle activation. While simultaneous timing may be viewed as an ideal way to exploit the elastic elements' ability to rapidly absorb energy, *in vivo* studies reveal that muscle activation happens before the arrival of energy input to the MTU. It remains an open question whether muscle pre-activation predisposes the MTU to further exploit its elastic elements to absorb peak power input, or shifts the MTU to an increased reliance on active muscle fascicle lengthening.

The purpose of this study was to mimic the *in vivo* muscle pre-activation strategy to investigate how it may influence the MTU's ability to deal with peak power input during an active stretch. We tested this idea by performing controlled *in situ* experiments to simply alter the onset timing of muscle activation to start simultaneously with or before the start of an active stretch applied to the MTU via a linear actuator (i.e. no pre-activation versus with pre-activation conditions). In this context, the experimental active MTU stretch with pre-activation condition would be analogous to the *in vivo* muscle pre-activation strategy observed during energy-absorbing activities. Inspired by previous work (Roberts and Azizi, 2010), we developed a simple metric (the peak power input decoupling ratio) to determine the extent to which power absorption demands of the muscle fascicles are decoupled from the total peak power input to the MTU during an active stretch. The decoupling ratio is calculated as one minus the ratio of muscle fascicle power input to the total MTU power input, such that higher values indicate a greater decoupling of power input, which can be attributed to the elastic elements' ability to rapidly absorb energy. In a scenario that yields a decoupling ratio of one, the muscle fascicles would only undergo active shortening or remain entirely isometric during an imposed stretch to the MTU and thus the elastic elements would stretch and absorb all the peak power input to the MTU. Conversely, a decoupling ratio of zero would indicate no contribution of elastic elements, but rather that muscle fascicles absorbed and dissipated all the peak power input to the MTU by undergoing active lengthening. However, muscle fascicles have been observed to undergo dynamic changes in shortening and lengthening while producing active force during an MTU stretch. These changes depend on the magnitude, rate and active duration of the MTU stretch (Griffiths, 1991; Roberts and Azizi, 2010), which will ultimately alter the elastic elements' contribution to decouple peak power inputs. Therefore, for a given MTU stretch, the peak power decoupling ratio is expected to fall along a spectrum between zero and one. So far, *in vivo* studies have revealed that muscle pre-activation can alter the initial contractile state of the muscle, i.e. increased force levels and shorter operating lengths, prior to the MTU undergoing an active stretch. However, it is not clear whether this pre-activation strategy predisposes the active MTU to a higher or lower peak power input decoupling ratio when compared with no pre-activation. Therefore, we tested the null hypothesis that the peak power decoupling ratio during an active MTU stretch would be similar between conditions of no pre-activation and with pre-activation. As a secondary analysis, we quantified changes in muscle fascicle behavior (shortening versus lengthening strains and timing) as the MTU undergoes an active stretch, which could give us insight into the mechanisms that either conserve or alter the peak power input decoupling ratio in the presence of muscle pre-activation.

**Table:** 

**List of symbols and abbreviations**	
COM	center of mass
*F*	force
*F*_0_	maximum isometric force
*L*	length of muscle fascicle
*L*_0_	optimal length of muscle fascicle
*L*_f_	final length of muscle fascicle
*L*_i_	initial length of muscle fascicle
LG	lateral gastrocnemius
*m*	mass of muscle–tendon unit
MTU	muscle–tendon unit
*P*_Fascicle_	power of muscle fascicle
*P*_MTU_	power of muscle–tendon unit
*P*_Peak_Fasicle_	peak power input of muscle fascicle
*P*_Peak_MTU_	peak power input of muscle–tendon unit
*V*_MTU_	velocity of muscle–tendon unit
*V*_Fascicle_	velocity of muscle fascicle
ε_L_	strain of muscle fascicle length

## MATERIALS AND METHODS

### Animals

Adult wild turkeys, *Meleagris gallopavo* Linnaeus 1758, were purchased from a local breeder and housed in the facilities within University Animal Care at the University of Arizona (mean±s.d. MTU mass 36.96±9.99 g, *n=*6). Our sample size of six was based on previous studies that performed similar *in situ* experiments that used five to seven animals (Roberts and Azizi, 2010; Griffiths, 1991). Using data from Roberts and Azizi (2010), a *t*-test was utilized to detect mean differences between two dependent groups (GPower 3.1). The following mean±s.d. values reported in Roberts and Azizi (2010) were: group 1, 557.6±57 W kg^−1^; group 2, 750±150 W kg^−1^, correlation=0.5. This yields an effect size of 1.47. Setting α=0.05 and β=0.20, a power analysis for a two-tailed *t*-test yields an objective sample size of 6 animals. Turkeys were maintained on a 12 h-light:12 h-dark cycle and provided with food and water *ad libitum*. This study was approved and conducted in accordance with the University of Arizona's Institutional Animal Care and Use Committee (IACUC).

### Surgical preparation and instrumentation

**Fig. 1.**
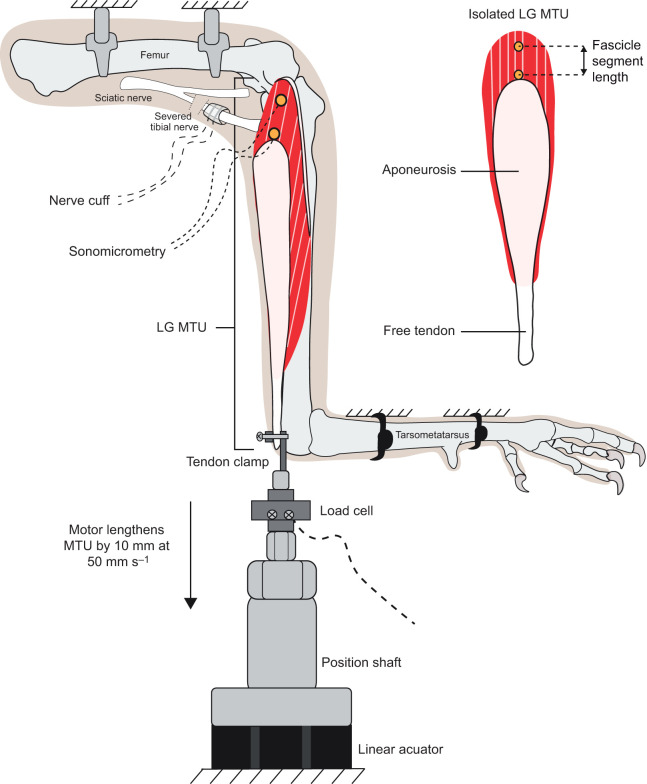
**Illustration of the *in situ* experimental setup using the turkey's lateral gastrocnemius muscle–tendon unit (LG MTU).** The entire right limb was fixed and secured at the femur and tarsometatarsus. The tibial nerve was severed from the sciatic nerve and instrumented with a custom, bipolar nerve cuff to stimulate the LG muscle. The LG muscle was instrumented with two sonomicrometry crystals to track a segment of the entire fascicle length. The LG tendon was severed from its insertion point and secured onto a tendon clamp that is in series with a load cell and a linear actuator that imposes a stretch to the entire LG MTU. Please note that during these experiments, the turkeys were under deep anesthesia while vital signs were continuously monitored (not illustrated for simplicity).

The *in situ* methods described here were performed on the right lateral gastrocnemius (LG) MTU of each turkey, as depicted in Fig. 1. The birds were first placed under deep anesthesia using inhalable isoflurane (1–3%) and laid on a surgical table with a heating pad to ensure maintenance of body temperature. A team of licensed veterinary technicians monitored the bird's vital signs (e.g. heart rate, body temperature, oxygen saturation, etc.) throughout the experiment.

For LG muscle stimulation, a custom-built, bipolar nerve cuff was placed on the tibial branch of the sciatic nerve. This nerve cuff was built from flexible tubing cut to ∼10 mm in length (Fisherbrand, ∼4.75 mm inner diameter×∼1.50 mm wall thickness) and instrumented with two silver wires ∼4 mm apart from each other while running through the middle of the tubing (0.254 mm bare, non-coated; AM Systems, Sequim, WA, USA; cat. no. 782500). A slit was cut horizontally through the length of the tubing so that the nerve cuff could be opened and wrapped around the nerve. Another small piece of tubing was added to the inside diameter so that the silver wires made maximal contact with the nerve. The silver wires acted as stimulating electrodes and were soldered onto longer, insulated wires. An alligator-to-BNC cable was used to connect the end wires of the nerve cuff to a voltage stimulator (Grass S48, Grass Technologies, West Warwick, RI, USA).

To implant the nerve cuff, an incision was made at the popliteal fossa to gain access to the nerve. A large retractor and blunt scissors were used to separate muscle, fascia and fat until the sciatic nerve was exposed. Custom glass probes with blunted edges were used to carefully separate the neurovascular bundle from the sciatic nerve and the fascia connecting the tibial and common peroneal nerve, which branch off from the sciatic nerve. The nerve cuff was then placed around the tibial branch of the sciatic nerve and secured by making a double square knot suture around the cuff using 3-0 suture (3-0 Securodox Plus, Polydioxanone Antibacterial Violet Monofilament Suture, 19 mm 3/8c Reverse Cutting; Securos Surgical, Fiskdale, MA, USA). Additionally, a double square knot suture was secured on the nerve just proximal to the nerve cuff and then sutured to a nearby muscle to prevent retraction of the nerve after severing it. Once secured, the tibial nerve was severed (proximal to the nerve cuff and suture) from the sciatic nerve to prevent stimulation of dorsiflexor muscles and to eliminate any contribution of spinal reflexes to muscle force output. To ensure that the nerve cuff was placed correctly, a twitch contraction was performed to elicit ankle plantar flexion. Once confirmed, the skin incision was closed with a continuous stitch using 3-0 suture.

To implant sonomicrometry crystals, a second incision was made on the limb, superficial to the middle of the LG muscle. Following this, we continuously applied saline solution, approximately every 5 min, onto the exposed muscle using a syringe to prevent drying. The muscle was carefully instrumented with two 2 mm sonomicrometry crystals to track length changes of the muscle fascicle (Sonometrics, London, ON, Canada). The first crystal was placed on the distal aspect of the superficial muscle fascicle, right above the apex of the aponeurosis. The second crystal was placed on the same muscle fascicle, but as far proximal as possible. Attempts were made to place crystals across the entire muscle fascicle; however, we found the strength of the signal was optimal at distances within 25 mm. Thus, if needed, we placed another crystal in between to ensure a high signal-to-noise ratio. In either case, the distance between a pair of crystals along a muscle fascicle is a measure of the segment of fascicle length. The percentage of the fascicle segment length captured relative to the entire fascicle length for each bird is reported in Table S1 (with an average of 68%). Each crystal was inserted into the muscle by first making a small incision using a 16-gauge hypodermic needle and then pushing the crystal into the muscle using a silastic tubing sleeve. The crystal was then secured into the muscle using a purse-string suture technique that ran through the muscle fascia and around the neck of the sonomicrometry crystal with 6-0 silk suture (Securosilk, Silk Black Braided Non-Absorbable Suture, 11 mm 3/8c reverse cutting; Securos Surgical). Once all the crystals were implanted and the quality of the signals was confirmed using an oscilloscope, the skin incision was then closed with an interrupted 3-0 suture.

To clamp the tendon to the motor, a third incision was made at the distal end of the LG MTU. Extra care was taken to separate the LG from the surrounding muscles (i.e. the medial gastrocnemius) by severing the LG tendon from its insertion point. The free tendon of the LG was then secured to a custom-built, stainless-steel tendon clamp with fine teeth to ensure a secure grip. We ensured that the LG MTU and linear actuator were along the same line of action by adjusting the side-to-side position of the motor. In addition, we accommodated any difference in vertical height between the LG MTU and the motor by placing extra towels underneath the bird. To help secure the bird's leg in place and prevent any movement artifacts during data collection, we secured the bird's femur bone onto two stainless steel clamps that were connected to a solid custom-built frame. This custom frame surrounded the surgical table and was built from T-slotted aluminium frames (80/20, LLC, Columbia City, IN, USA). The tarsometatarsus was also secured onto a cross-bar of the custom surgical table frame using heavy-duty cable ties to configure the ankle at a fixed 90 deg angle.

### Experimental protocol during data collection

Maximal stimulation of the LG was first determined through a series of twitches using square wave pulses set to 0.2 ms duration. The starting voltage was set to 3 V and increased by 1 V until no further increase in force occurred (i.e. maximum twitch) for each bird. This voltage was then multiplied by 1.5 and used as the supramaximal stimulation for the rest of the experiment (average 14 V). Compared with previous reports (Roberts and Azizi, 2010; Konow et al., 2012), it is likely that our higher voltage stimulation stems from the design of our nerve cuff with a relatively greater distance between electrodes and different wire diameter, and possibly from the larger muscle masses used in this study. The frequency of stimulation that elicited a maximum tetanus force and reached a plateau was determined for each bird and used for the rest of the experiment. The frequencies used in this study ranged between 50 and 75 pulses s^−1^ for all birds. Next, a series of fixed-end, tetanic contractions at various fascicle lengths were performed to produce a tetanic force–length curve from the muscle fascicle segment length measured by the sonomicrometry and the force measured by the load cell in series with the linear actuator. We performed these tetanic contractions with a stimulation train duration set to a minimum of 200 ms to ensure the force reached a plateau. A minimum of 5 min of rest was given between contractions to minimize fatigue. During data collection, we quickly determined an estimated optimal fascicle length based on the muscle fascicle segment length that was estimated to produce peak active force to help determine the initial fascicle resting length for the experimental conditions (i.e. no pre-activation and with pre-activation). These estimations were made because of time constraints and technical limitations of data collection in different software. Please note that formal analysis of the tetanic force–length curve was performed after data collection and is described in detail in ‘Measurements and data analysis after data collection’, below.

To determine the duration of pre-activation for each bird, we chose to take a percentage of the time to peak tetanus force to determine the absolute duration (ms) within a biologically relevant time window (i.e. 0–100% time to peak tetanus force). A fixed-end tetanus contraction trial that started at ∼5 N of passive force was used to determine the time to peak tetanus force. We then calculated 10% of the time to reach peak tetanic force to determine the duration of pre-activation for each bird in this study. This percentage was chosen as part of a protocol for a larger experiment aimed at probing the limits across the entire biologically relevant time range (D.V. and C.J.A., unpublished data). The pre-activation duration (i.e. the duration of muscle activation before MTU stretch) used in this study ranged from 30 to 55 ms, with an average of 42 ms (Table S1). It is important to note that animals, including humans, use a range of pre-activation durations (e.g. 30–400 ms; Griffiths, 1989; Santello and McDonagh, 1998; Konow and Roberts, 2024), but the underlying mechanism that determines these durations is not fully understood. For this study, however, the pre-activation durations used are biologically relevant and can be considered short.

For the experimental trials, we first performed the no pre-activation trial and then the trial with pre-activation to avoid any early damage that may be induced during a lengthening contraction. For each experimental trial, the MTU was first adjusted to a resting muscle fascicle segment length (a real-time measure by the sonometric software) that was estimated to be at the muscle fascicle's optimal length. For each bird, the resting fascicle segment length was kept the same for the conditions of no pre-activation and with pre-activation. Thus, the fascicle operating length (once expressed relative to optimal length after formal analysis) was kept the same for both conditions for a given bird. The fascicle operating lengths during resting baseline are reported in Table S2 for all birds. The timing of muscle stimulation and the imposed stretch by the linear actuator onto the MTU were adjusted and controlled by a Grass voltage stimulator. The onset timing of muscle stimulation was manipulated to start (1) simultaneously with or (2) before the linear actuator imposed a stretch on the entire MTU, i.e. no pre-activation versus with pre-activation conditions. For the pre-activation condition, the duration of muscle activation before the start of MTU stretch was based on the 10% time to peak tetanic force (Table S1). For both trials, the muscle was stimulated supramaximally for a total stimulation duration set to a minimum of 500 ms to ensure the muscle fascicles were active during the entire MTU stretch and as part of an experimental protocol for a larger study. In this study, the total duration of the ramp stretch occurred within 200 ms and therefore we focus our analysis within this active MTU stretch. The linear actuator stretched the MTU at a fixed magnitude of 10 mm at a rate of 50 mm s^−1^ for both trials, which gives rise to a total MTU stretch duration of 200 ms. Given previous reports (Griffiths, 1991; Roberts and Azizi, 2010; Konow and Roberts, 2015), these MTU stretch parameters can be considered within the bounds of being biologically relevant. While the relative magnitude of this 10 mm stretch can vary between birds as a result of morphological differences (Table S1), the same stretch was applied to the MTU for both experimental trials and thus each bird is their own control measure. The motor dwell delay was set to 1 s to allow for the MTU to remain stretched at this final 10 mm position (analogous to an isometric contraction). Following this dwell time, the motor moved the MTU back to its original starting position at the same rate (50 mm s^−1^) and duration (200 ms). A minimum of 5 min was given for rest between experimental trials to minimize fatigue. After completion of the trials, the muscle was set close to the reference optimal length based on the muscle fascicle segment (measured via sonomicrometry) while measurements of the entire fascicle length were made with calipers (Neiko 01407A Digital Caliper). These measures were recorded to adjust for the entire fascicle length during data analysis. Once all measures were taken, the bird was put down by intravenous injection of an overdose of Beuthanasia-D (pentobarbital sodium). The LG MTU was then carefully dissected from the limb and weighed.

### Equipment and data collection parameters

The custom-built tendon clamp was attached to the linear actuator (Kollmorgen, Radford, VA, USA; EC3 Series Electrical Cylinders) and in series with a tension load cell (Omega Engineering Inc., Stamford, CT, USA; Model: LCM703-250) using a stainless-steel screw adapter. This adapter provided a rigid setup to eliminate system compliance during data collection. The linear actuator was secured onto the custom frame surrounding the surgical table and locked in place prior to any experimental trials. The linear actuator was powered and controlled by an AKD drive (Kollmorgen, Radford, VA, USA) and Kollmorgen WorkBench software. The load cell was powered and conditioned by an amplifier (Omega Engineering Inc.; Model: DMD-465WB). Analog signals from the linear actuator and load cell were collected via a National Instruments Data Acquisition board (NI cDAQ-9178; National Instruments Corp., Austin, TX, USA) using a differential input signal setup. These analog signals were then converted into a digital signal on a laptop (Dell Inc., Round Rock, TX, USA; Dell Precision 7710) sampled at 10,000 Hz in IgorPro (WaveMetrics, Portland, OR, USA). A high sampling rate was chosen to simultaneously record the muscle stimulation signal output from the Grass voltage stimulator.

All implanted sonomicrometry crystals were connected to the Sonometrics DS3-8 hardware and grounded by placing the alligator clip ground cable between the muscle and skin. Digital signals were recorded in the SonoLabDS3 data acquisition software with the following settings: inhibit delay set to 4.02 mm, transmit pulse set to 406.25 ns and sampling rate set to 508 Hz. While this sampling rate is lower compared with previously reported rates of sonomicrometry in muscles, we found it to be adequate for our study. At higher frequencies (e.g. 1000 Hz), we observed high noise in our sonometric signals, which we believe arose from the linear actuator being powered on. The quality of the receiver and transmitter transducer signals was consistently checked via an oscilloscope (Tektronix, Inc., model: TBS 1072B-EDU) to ensure that the first peak was being tracked. An external trigger box was used to send a synchronized signal (0–3.3 V) simultaneously to the analog input within the Sonometrics hardware and within the NI DAQ board for synchronization of all signals post-processing.

For the timing of muscle stimulation and MTU stretch, a Grass voltage stimulator with two stimulator units was used (Grass S48, Grass Technologies). The start trigger of one stimulator unit was used to output muscle stimulation parameters (voltage, train, duration) to the nerve cuff and thus the LG muscle. At the same time, an output trigger from this unit was sent to the NI DAQ system to start data collection in IgorPro via a digital trigger, and into the second stimulator sync-in port, which controlled the timing of the stretch by the motor. The delay dial on the second stimulator unit was used to control the timing of MTU stretch relative to muscle activation by sending a 24 V trigger to the linear actuator to stretch the MTU at the specified magnitude and rate (10 mm at 50 mm s^−1^) as set in the Kollmorgen WorkBench software. The signal for stimulation to the muscle was set up so that the Grass stimulator sent a synchronization analog output signal (0–5 V) that could be recorded in a designated NI DAQ system channel.

### Measurements and data analysis after data collection

All data were analyzed in Igor Pro (WaveMetrics, Portland, OR, USA) using custom code. Data recorded in the SonoLabDS3 software were exported and then imported into the Igor Pro experiment. The sonometric signals and those collected by the NI DAQ system in Igor Pro were then adjusted by taking into account the synchronized signal recorded by both systems. The sonometric signals were first inspected for any high-frequency spikes or outliers in the signal, which were corrected for by either using a median smoothing filter set to a threshold difference of 0.2 mm to remove single points or manually replacing them with NaN values. Level shifts, if any, were corrected for by using a custom Igor Pro function that assumes the level shift should be corrected to a similar slope to the two points preceding and/or following the level shift. Using previously established smoothing techniques (Arellano et al., 2016), the sonometric signals were filtered using a smoothing spline interpolation with a smoothing factor of 1 and a standard deviation that was quantified from a 500 ms resting baseline and adjusted by a multiplier (0.2–2.5) that was determined upon visual inspection to best fit the original signal without over-smoothing or causing significant time distortions. An example of filtered data is provided in Fig. S1D. All sonometric measurements were multiplied by the ratio of the entire muscle fascicle length to the measured fascicle segment length that was recorded during data collection to estimate the total muscle fascicle length measurements (which we refer to as muscle fascicle length). The motor analog signals were converted to position signals (mm) using a conversion factor provided by the Kollmorgen software. The load cell signals were converted to force (N) using a conversion factor determined from our calibration process using known weights ranging from 0 to 250 lbs (0 to ∼113 kg). Force values were adjusted by subtracting any baseline offset measured from the load cell prior to tendon clamping. Baseline offsets of the load cell were checked before and after experiments to ensure no significant drift occurred during the experiment. Motor position and force signals were then filtered using similar smoothing techniques to those previously described, with a smoothing factor of 1 and a standard deviation that was quantified from a 500 ms resting baseline and adjusted by a multiplier that best fitted the data (i.e. 0.825–2.85 multiplier for motor position and 0.75 multiplier for force signals). An example of filtered data is provided in Fig. S1B,C.

In order to calculate instantaneous power measurements, all signals were resampled to 500 Hz using a linear interpolation method to match the lower sampling rate of the sonometric signals. The muscle fascicle length and motor position signals were then differentiated using the central differences method to output fascicle velocity and MTU velocity, respectively. The velocities were then multiplied by −1 so that negative values signify lengthening velocity and positive values signify shortening velocity. Instantaneous MTU power (*P*_MTU_) was calculated as the product of MTU velocity (*V*_MTU_; m s^−1^) and MTU force (*F*; N) as shown in Eqn 1:
(1)

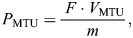
where *m* is mass of the muscle–tendon unit. Instantaneous fascicle power (*P*_Fascicle_) was calculated from the product of fascicle velocity (*V*_Fascicle_; m s^−1^) and MTU force (N) as shown in Eqn 2:
(2)

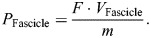
Power values were normalized to MTU mass (kg) to express power in W kg^−1^ units (Eqns 1 and 2). Positive power values are referred to as power outputs, while negative power values are referred to as power inputs. Peak power inputs (*P*_Peak_) of the muscle fascicles and MTU were taken during the MTU stretch and then expressed as a peak power input decoupling ratio, as shown in Eqn 3:
(3)

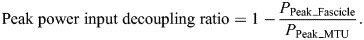
The decoupling, which is the discrepancy in the peak power input between the muscle fascicles and MTU, can be attributed to the elastic elements' ability to rapidly absorb energy. By this calculation, a higher decoupling ratio signifies a greater decoupling of peak power inputs between muscle fascicles and the MTU, indicating a greater reliance on elastic elements to rapidly absorb energy. In contrast, a lower decoupling ratio signifies a lower decoupling of peak power inputs, indicating a greater reliance on muscle fascicles to rapidly absorb energy.

The tetanic force–length curve was constructed for each bird from a series of fixed-end, tetanic contractions for a range of fascicle lengths at maximal stimulation (similar methods to those in Holt and Azizi, 2014; Schwaner et al., 2024; Arellano et al., 2016). For each fixed-end contraction, the active force was calculated by subtracting the initial passive force from the total force. The maximal active force and its corresponding final fascicle length were determined for each fixed-end contraction to construct the active force–length curve. Additionally, the passive force–length curve was constructed by taking the passive force and its corresponding fascicle length for each fixed-end contraction prior to stimulation. Thus, this relationship characterizes the passive properties of muscle fascicles and not the MTU (Mendoza and Azizi, 2021). A third-order polynomial was fitted to the active force data to obtain maximum isometric force (*F*_0_) and optimal fascicle length (*L*_0_). It should be noted that *L*_0_ is determined here from the estimated total muscle fascicle length measures (where sonometric signals were adjusted to the entire fascicle length). During post-processing, we were unable to construct a force–length curve for one bird because of not having enough data points in the descending limb for a good polynomial fit. Thus, we could not include this bird as part of the analysis where *L*_0_ and *F*_0_ are needed, but we were able to include this bird in the analyses of peak power inputs.

The time-series data for muscle fascicle length (for conditions of no pre-activation and with pre-activation) were normalized by dividing instantaneous muscle fascicle length (*L*) by optimal length (*L*_0_) to obtain relative fascicle length as shown in Eqn 4:
(4)

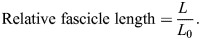
Similarly, the time-series data for force were normalized by dividing the instantaneous MTU force (*F*) by maximum isometric force (*F*_0_) to obtain relative force as shown in Eqn 5:
(5)

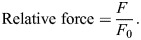
Strain values for fascicle length (ε_L_) were calculated by taking the final (*L*_f_) and initial (*L*_i_) fascicle length measures for a given period and then dividing by optimal fascicle length (*L*_0_) as shown in Eqn 6:
(6)

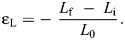
Positive and negative strain indicate fascicle shortening and lengthening, respectively.

**Fig. 2.**
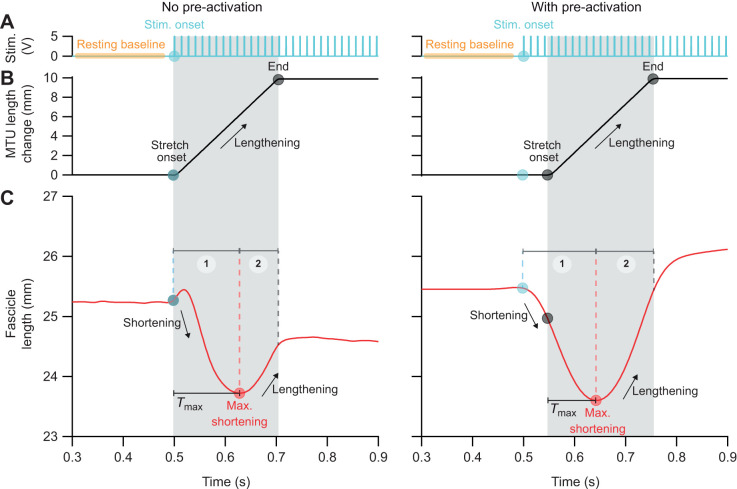
**Example time-series data demonstrating the muscle fascicle undergoing a transition from active shortening to active lengthening during an active MTU stretch (*n*=1).** (A) Onset muscle activation timing (blue circle) was manipulated via a Grass stimulator to occur either (1) simultaneously with or (2) before a stretch applied to the MTU via a linear actuator (i.e. no pre-activation condition versus with pre-activation condition). Supramaximal stimulation was recorded as a 0–5 V output from the Grass stimulator. (B) In both conditions, the same stretch was applied to the MTU while the muscle remained activated throughout the entire stretch (shaded gray region), with a total ramp stretch duration of 200 ms. (C) The fascicle transition point was identified as the point of maximal fascicle shortening (red circle) during the stretch. Phase 1 consists of the time between stimulation onset and maximum fascicle shortening point, while phase 2 consists of the time between maximum fascicle shortening point and the end of MTU stretch. Note that net fascicle strains were extracted during these phases. The time to the maximum fascicle shortening point, relative to the onset of MTU stretch (*T*_max_), was also extracted for analysis.

For our secondary analysis, we observed that under both conditions, the muscle fascicles underwent a shortening period and then transitioned to a lengthening period during the MTU stretch. We identified this fascicle transition point which corresponds to maximum fascicle shortening and thus partitioned the analysis into two phases to better understand the dynamic behavior underlying MTU stretch. As shown in Fig. 2, phase 1 is the period from onset of muscle activation to the point of maximum fascicle shortening, and phase 2 is the period from the point of maximum fascicle shortening to the end of MTU stretch. Fascicle strain values were extracted during these phases. Net fascicle strain was extracted from the period of onset muscle activation to the end of MTU stretch. Furthermore, we quantified the time to the maximum fascicle shortening point relative to the onset of MTU stretch. Given that the MTU was stretched at a fixed rate and magnitude (i.e. stretch for 10 mm at 50 mm s^−1^), the total time for the entire MTU stretch was 200 ms for both conditions. Therefore, absolute measures in milliseconds were compared between conditions.

### Statistical analysis

For all dependent variables, the assumption of normality was first assessed by the Shapiro–Wilk test. If the assumption of normality was met (*P*>0.05) for a given variable, then we ran the standard paired *t*-test. Otherwise, we ran the non-parametric Wilcoxon signed-rank test. These statistical tests are appropriate and account for individual differences as the same muscle from the same bird was used for both conditions of no pre-activation and with pre-activation. All statistical tests were run with an alpha level of 0.05 (IBM SPSS Inc., Chicago, IL, USA).

To assess whether pre-activation changed the initial state of the MTU as intended by the experimental design, we performed the appropriate statistical test (i.e. the parametric paired *t*-test or the non-parametric Wilcoxon signed-rank test) between the no pre-activation and with pre-activation conditions, and assessed the one-tailed *P*-value for initial relative force and fascicle length strain at the start of MTU stretch relative to the onset of muscle activation. To test our hypothesis, we performed a paired *t*-test to compare the means of the peak power decoupling ratios between no pre-activation and with pre-activation conditions and assessed the two-tailed *P*-value. The effect size for the peak power inputs and decoupling ratio is reported using Cohen's *d*. For our exploratory analysis, we performed the appropriate statistical test (i.e. the parametric paired *t*-test or the non-parametric Wilcoxon signed-rank test) among the variables of interest between the two conditions with a two-tailed *P*-value. All data underlying the descriptive and inferential statistics are provided in Tables S1–S7. The summary values are reported as means±s.d. in the text, with values for the condition of no pre-activation mentioned first, followed by values for the condition with pre-activation.

## RESULTS

### Initial MTU conditions prior to MTU stretch

**Fig. 3.**
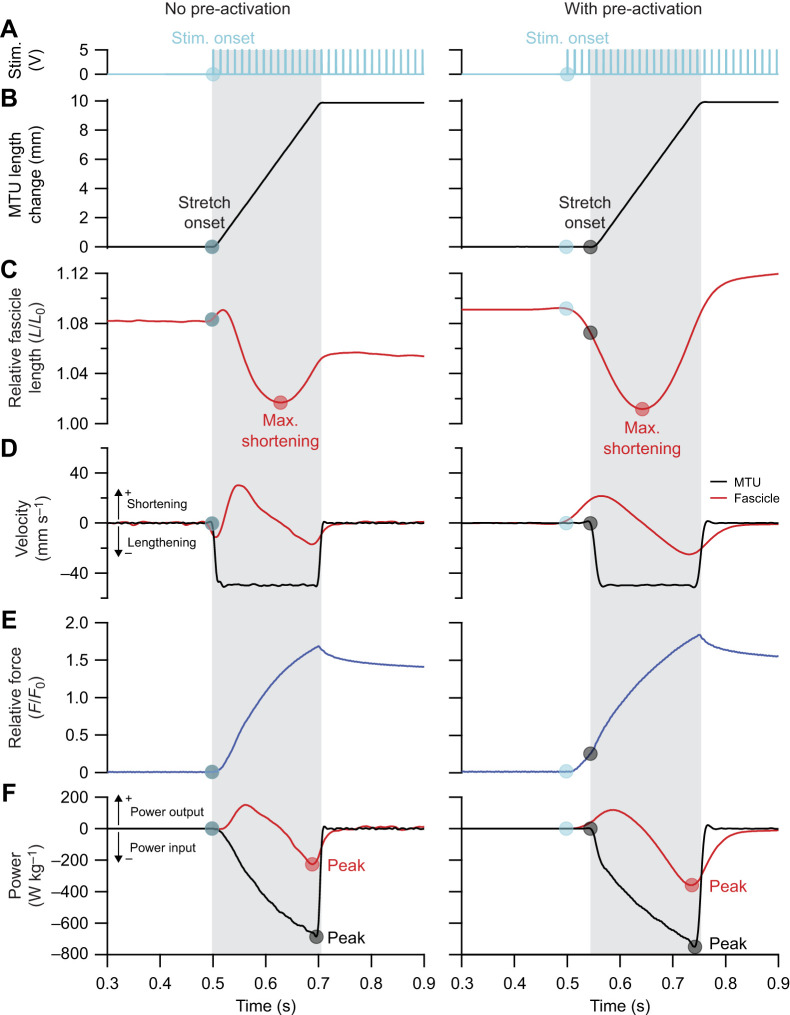
**Representative time-series data for an active stretch to the LG MTU reveals that muscle fascicles absorb greater peak power input with pre-activation (*n*=1).** (A) Onset muscle activation timing (blue circle) was manipulated to occur simultaneously with or before a stretch applied to the MTU (i.e. no pre-activation versus with pre-activation condition). (B) In both conditions, the same stretch (10 mm at 50 mm s^−1^) was applied to the LG MTU via a linear actuator while the muscle remained activated throughout the entire stretch. The shaded gray region indicates the period of MTU stretch. (C) The muscle fascicle reached a point of maximum shortening during the MTU stretch. (D) Position signals from the linear actuator and muscle fascicle length measures were differentiated to calculate velocity. (E) Force was recorded via a load cell in series with a linear actuator and the LG MTU. (F) Instantaneous power was calculated from the product of velocity and force. Peak power inputs of the muscle fascicle (red circle) and MTU (black circle) were extracted to calculate the peak power input decoupling ratio for each condition. Note that with pre-activation, the muscle fascicle reached a point of maximum shortening earlier into the stretch, allowing more time to actively lengthen and produce greater force due to force–velocity effects.

On average, the relative fascicle length during resting baseline was set to 1.10±0.15 *L*/*L*_0_ for both conditions (Table S2; minimum: 0.93 *L*/*L*_0_, maximum: 1.33 *L*/*L*_0_; *n=*5). Initial relative force at the start of MTU stretch was significantly greater with pre-activation compared with no pre-activation (0.03±0.01 *F*/*F*_0_ versus 0.31±0.07 *F*/*F*_0_; paired *t*-test, one-tailed, *P*<0.001; *n=*5). Muscle fascicles underwent significant shortening strains at the start of MTU stretch relative to the onset of stimulation with pre-activation compared with no pre-activation (*z*=−2.023, Wilcoxon signed-rank test, one-tailed, *P*=0.022; *n=*5). Representative data in Fig. 3 highlight the changes induced by pre-activation compared with no pre-activation.

### Peak power input and decoupling ratio during MTU stretch

**Fig. 4.**
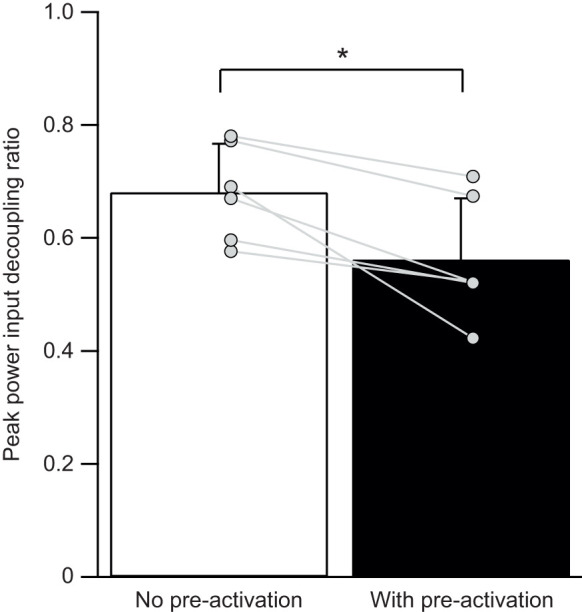
**An active MTU stretch with pre-activation reduces the peak power input decoupling ratio.** This finding indicates that muscle fascicles were required to absorb a greater percentage of the total peak power input. Each line segment represents an individual LG MTU, highlighting that all MTUs showed a reduction in the decoupling ratio during a stretch that occurred with pre-activation (mean±s.d., 0.68±0.09 versus 0.56±0.11; paired *t*-test, two-tailed, *P*=0.015; Cohen's *d*=1.49; *n*=6). *P<0.05.

An active MTU stretch with pre-activation had greater MTU peak power input compared with no pre-activation (−579±107 W kg^−1^ versus −632±119 W kg^−1^; paired *t*-test, two-tailed, *P*=0.004; Cohen's *d*=2.03; *n*=6). Muscle fascicles experienced greater peak power input with pre-activation compared with no pre-activation preceding an active MTU stretch (−182±48 W kg^−1^ versus −278±87 W kg^−1^; paired *t*-test, two-tailed, *P*=0.015; Cohen's *d*=1.47; *n=*6). The peak power decoupling ratio was significantly lower in the pre-activation condition compared with the no pre-activation condition (0.68±0.09 versus 0.56±0.11; paired *t*-test, two-tailed, *P*=0.015; Cohen's *d*=1.49; *n*=6; Fig. 4).

### Key features that dynamically alter peak power input during MTU stretch

Muscle fascicles reached a point of maximum shortening earlier into the MTU stretch with pre-activation compared with no pre-activation (110±19 ms versus 76±24 ms; paired *t*-test, two-tailed, *P*=0.001; *n*=6). At this maximum shortening point, the relative fascicle length was shorter with pre-activation (1.03±0.08 *L*/*L*_0_ versus 1.00±0.08 *L*/*L*_0_; paired *t*-test, two-tailed, *P*=0.023; *n*=5). The relative force at this maximum shortening point was not significantly different between conditions (0.93±0.25 *F*/*F*_0_ versus 0.93±0.24 *F*/*F*_0_; paired *t*-test, two-tailed, *P=*0.975; *n*=5). From the period of onset stimulation to the point of maximum fascicle shortening, muscle fascicles underwent greater shortening strain with pre-activation compared with the no pre-activation condition (*z=*−2.023; Wilcoxon signed-rank test, two-tailed, *P=*0.043, *n*=5). When compared with the no pre-activation condition, muscle fascicles underwent greater lengthening strain from the period of maximum fascicle shortening to the end of MTU stretch (−0.04±0.01 versus −0.07±0.02; paired *t*-test, two-tailed, *P*=0.011). In addition, the relative force at the end of the MTU stretch was higher in the pre-activation condition (1.31±0.20 *F*/*F*_0_ versus 1.44±0.23 *F*/*F*_0_; paired *t*-test, two-tailed, *P=*0.023). Yet, the net fascicle strain at the end of the MTU stretch was not significantly different between conditions (*z*=−0.14; Wilcoxon signed-rank test, two-tailed, *P*=0.893).

## DISCUSSION

Our findings reveal that an active MTU stretch with pre-activation reduces the peak power input decoupling ratio when compared with an active MTU stretch with no pre-activation (Fig. 4). This leads us to reject the null hypothesis that the peak power input decoupling ratio during an active MTU stretch would be similar between no pre-activation and with pre-activation conditions. The lower decoupling ratio indicates that muscle fascicles absorbed a greater percentage of the total peak power input of the MTU, signifying a decreased contribution of elastic elements to rapidly absorb energy. We also found that an active MTU stretch with pre-activation allowed the MTU to absorb greater total peak power input. In the condition of no pre-activation, i.e. when onset of activation simultaneously occurred with onset of MTU stretch, the MTU absorbed an average peak power input of −579 W kg^−1^. This value falls within the range of previously reported values of LG MTUs using simultaneous timing of MTU stretch with onset activation, where stretches of 40 mm s^−1^ (at 6 mm) and 60 mm s^−1^ (at 8 mm) elicit average peak power inputs of −453 and −769 W kg^−1^, respectively (values extracted from fig. 4 in Roberts and Azizi, 2010, using WebPlotDigitizer software). However, when comparing the active MTU stretch that occurred with pre-activation, the MTU experienced an increase in average peak power input of −632 W kg^−1^, despite undergoing the same rate and magnitude of MTU stretch as the no pre-activation condition. Thus, while pre-activation reduces the reliance on elastic elements to decouple peak power inputs into the MTU, our findings reveal an unexpected trade-off in which active lengthening of muscle fascicles allows the MTU to deal with a greater total peak power input.

During the active MTU stretch, for both conditions of no pre-activation and with pre-activation, muscle fascicles reached a point of maximum shortening during the stretch and then transitioned to lengthening for the remainder of the stretch. Therefore, during the early phase of the MTU stretch, muscle fascicles were actively shortening, which indicates that the elastic elements were effective in decoupling instantaneous peak power input. However, in the condition with pre-activation, muscle fascicles achieved a greater shortening strain, which facilitated a greater lengthening strain during the late phase of the stretch. This suggests that although the elastic elements were effective in decoupling power input during the early phase of the stretch, their contribution to decouple peak power input later into the stretch was reduced as muscle fascicles underwent greater active lengthening. Furthermore, the net fascicle strain achieved at the end of the MTU stretch relative to the start of muscle activation did not differ between conditions. Our findings regarding fascicle strain are consistent with a pre-activation strategy employed by toads *in vivo*, where they shorten their muscle fascicles prior to landing to help accommodate the greater lengthening strain needed for increased jumping distances while constraining them to a maximal operating length (Azizi and Abbott, 2013). However, the key feature revealed in our analysis is that with pre-activation, muscle fascicles transitioned from active shortening to lengthening earlier into the stretch and, consequently, muscle fascicles spent more time actively lengthening during MTU stretch. Therefore, pre-activation alters the timing of the dynamic behavior of muscle fascicle shortening and lengthening, which predisposes muscle fascicles to greater lengthening strains and durations.

Another key feature to appreciate is that the muscle fascicles were undergoing active lengthening during MTU stretch and therefore the muscle's intrinsic force–velocity effects in the lengthening domain were revealed with our experimental design. In our study, the muscle was stimulated during the entire duration of MTU stretch for both conditions of no pre-activation and with pre-activation. This differs from a previous experimental study where stimulation to the muscle ceased during the applied stretch to the MTU (Roberts and Azizi, 2010) and therefore the muscle's force–velocity effects in the lengthening domain could not be fully appreciated. In our study, we found that force continued to rise as fascicles underwent active lengthening during MTU stretch, revealing that muscle fascicles were exploiting the muscle's force–velocity property in the lengthening domain. This phenomenon is characterized by the muscle fascicle's ability to produce rapid and high forces (i.e. greater than *F*_0_) when undergoing active lengthening compared with active shortening (Katz, 1939; McMahon, 1984). In Fig. 3, one can see that in both experimental conditions, the MTU reached forces greater than peak isometric force during stretch, which owes to the muscle fascicles operating in the lengthening domain of their force–velocity curve. However, as pre-activation altered the timing of muscle fascicle length behavior, the muscle fascicles were driven to a dynamic state that further exploited the force–velocity effects in the lengthening domain. Further exploitation of force–velocity effects is supported by the observation that the muscle fascicles generated greater forces at the end of the MTU stretch with pre-activation (1.31 versus 1.44 *F*_0_) and thus facilitated the MTU's ability to absorb greater peak power input.

Other studies have revealed the lengthening force–velocity effects when a stretch is applied to a muscle, but only after it has been maximally stimulated and thus reached a maximal isometric force before the stretch is applied (Rassier and Herzog, 2002; Lieber and Fridén, 1993). This contrasts with our experimental design, where the imposed stretch occurred before reaching a maximal isometric force, with average force levels at the time of stretch being 0.03 *F*_0_ for no pre-activation conditions and 0.31 *F*_0_ with pre-activation conditions. Even though the muscle did not reach maximal isometric force before the applied stretch, the muscle still exploited its intrinsic force–velocity properties in the lengthening domain during the stretch. This may have occurred not only because the MTU was active during the entire stretch but also because of the magnitude of stretch applied here, which was 10 mm. If the stretch magnitude was shorter (e.g. 5 mm), then possibly the muscle fascicles would have not been predisposed to active lengthening during the MTU stretch. However, a stretch magnitude of 10 mm of the LG MTU has been observed *in vivo*, where turkeys undergo significant ankle and knee flexion to absorb energy from a landing height that is considered a high energy-absorbing task (Konow and Roberts, 2015). Thus, the intent behind the experimentally applied stretch was to simulate the demand for the LG MTU to rapidly absorb energy, analogous to turkeys having to meet the high energy-absorbing demands of drop landing *in vivo*. Therefore, our data suggest that a pre-activation strategy *in vivo* may allow muscle fascicles to exploit their lengthening force–velocity effects, especially under dynamic conditions where the MTU must deal with greater rates of energy demand.

Our study provides new insights into a pre-activation strategy, but there are intriguing intricacies to be further explored in future experiments. A limitation of this study was the variability in the initial relative fascicle length (minimum: 0.93 *L*/*L*_0_ and maximum: 1.33 *L*/*L*_0_) prior to active MTU stretch, which likely influenced the absolute values of peak force and peak power input observed among individuals. However, the initial relative fascicle length was the same for both experimental conditions for a given individual (see Table S2) and thus it does not change the interpretation of our key findings regarding the decreased peak power input decoupling ratio and the increase in total peak power input of the MTU when comparing the condition of stretch with pre-activation to the condition of no pre-activation. Furthermore, despite the variability in initial relative fascicle length, all individuals experienced the same length behavior of muscle fascicles during MTU stretch (i.e. initial active shortening followed by active lengthening) and increased total peak power input of the MTU with pre-activation (see Table S4). In future experiments, it would be worthwhile to explore whether the MTU can optimize a muscle's pre-activation strategy to generate greater forces and increase its peak power input capacity by simply altering the muscle fascicle's initial length to operate on the descending limb (greater than *L*_0_), the ascending limb (less than *L*_0_) or the plateau (at *L*_0_) of its force–length curve. While we would expect muscle force to be influenced by force–length effects, we would also expect that an intricate interaction takes place between changes in the relative stiffness of elastic elements and changes in the force–velocity effects when operating along different regions of the force–length curve, which will likely limit or maximize active fascicle lengthening. Given that toads start at the plateau of their force–length curve prior to a pre-activation strategy in preparation for landing after a jump (Azizi and Abbott, 2013), we suspect that the MTU could be optimized to generate greater forces when initially operating at the plateau of their force–length curve.

Another intriguing aspect to consider is how changes in muscle activation timing could alter the reliance on muscle fascicles versus elastic elements. In this study, we focused on a subset of data from a larger experiment (D.V. and C.J.A., unpublished data), but our future work will investigate how increasing the duration of muscle pre-activation may alter the MTU's ability to deal with energy absorption. We hypothesize that increasing the duration of muscle pre-activation will likely predispose the muscle fascicles to greater active fascicle lengthening strains during an MTU stretch, which would likely exploit the lengthening force–velocity effects by allowing the muscle to produce more force and thus would increase the MTU's capacity to deal with greater total peak power input. This may underlie the explanation for the *in vivo* observation of animals increasing the duration of muscle pre-activation with increasing landing heights (e.g. Konow and Roberts, 2024; Santello and McDonagh, 1998), as it is anticipated that the MTU would be required to generate greater forces and thus deal with greater peak power inputs. Furthermore, we remain intrigued as to how activation and deactivation dynamics during MTU stretch may alter the reliance between muscle fascicles and elastic elements. In future work, we intend to study how the duration of muscle activation during MTU stretch could alter the MTU's ability to deal with energy absorption and shift the reliance between fascicles and elastic elements. In our study, the muscle was stimulated throughout the entire MTU stretch, and thus the active muscle fascicle lengthening contributed to the force rise during MTU stretch. However, if stimulation ended earlier, such as half or a quarter of the way into the MTU stretch, then we suspect that this shorter active duration during MTU stretch would likely prevent muscle fascicles from active lengthening, which would likely decrease force output and thus decrease the capacity of the total peak power input that the MTU can deal with.

Lastly, we acknowledge several limitations in our study. For our metric of the peak power input decoupling ratio, the decoupling ratio was calculated as one minus the ratio of muscle fascicle peak power input to the total MTU peak power input. Therefore, the discrepancy in the peak power input between the muscle fascicles and the MTU can be attributed to the elastic element's ability to decouple by stretching and storing elastic energy. However, we recognize that there may be other mechanisms underlying this decoupling such as architectural gearing due to dynamic changes in pennation angle (Azizi et al., 2008; Azizi and Roberts, 2014) as well as muscle shape change influencing aponeurosis behavior to modulate longitudinal MTU stiffness (Azizi and Roberts, 2009; Arellano et al., 2016, 2019). While these mechanisms may play a role and likely alter the peak power input decoupling ratio, these factors would not change our overall key finding that there was an increased reliance on active fascicle lengthening and an increase in the total peak power input of the MTU during an MTU stretch with pre-activation. Another limitation of our study is that we did not randomize the condition order; rather, we performed the condition of no pre-activation first followed by the condition with pre-activation, which may have biased our results. For instance, it is well established that eccentric contractions predispose muscle fascicles to damage (Lieber and Fridén, 1993, 2002; Proske and Morgan, 2001) and that such damage correlates with a reduced force capacity (Warren et al., 1993; Allen, 2001). Therefore, we reason that our experimental trial order could have biased our results by underestimating the forces observed in the second condition (i.e. with pre-activation), presuming muscle damage occurred in the first condition of stretch (i.e. no pre-activation). Yet, this underestimation would not change the trends we observed in this study as our data reveal that significantly greater peak forces are achieved at the end of MTU stretch with pre-activation. *In situ* studies allow for highly controlled experiments, but future work should continue to unravel the intricate interactions that can arise between muscle fascicles and elastic elements that allow for the MTU to deal with various energy demands *in vivo*.

In summary, our findings reveal a unique trade-off that exists when muscles are pre-activated prior to an active MTU stretch. A reduction in the peak power input decoupling ratio suggests a decreased reliance on the elastic elements to absorb and decouple peak power inputs as the muscle fascicles were predisposed to greater active lengthening strains and durations. However, the increased reliance on muscle fascicles to actively lengthen during an MTU stretch allows the MTU to generate greater force, which increases the capacity of the MTU to deal with greater peak power input. Functionally, a greater capacity for peak power input of the MTU is needed to match the high energy demands to perform a variety of locomotor tasks, such as landing, sprinting and downhill running. In the context of drop landings, the higher the drop height, the greater the energy demand. This is supported by direct evidence from *in vivo* experiments where turkeys experience greater peak power input of the LG MTU with increased drop height (Konow and Roberts, 2015). Overall, the insights gained from our experiment and those observed *in vivo* suggest that a simple, neural control strategy of pre-activation can be exploited to prime the MTU to rely on active muscle fascicle lengthening to favor a greater rate of energy demand, despite reducing the elastic element's contribution to decouple peak power inputs.

## Supplementary Material

10.1242/jexbio.251324_sup1 Supplementary information
