# Supplementary material for: In situ muscle pre-activation shifts the lateral gastrocnemius muscle–tendon unit to rely on active fascicle lengthening to absorb peak power input
Source: J Exp Biol. 2026 Mar 10;229(5):jeb251324. doi: 10.1242/jeb.251324 (PMC13006518; doi:10.1242/jeb.251324)
Supplement: Supplementary information [file jexbio-229-251324-s1.pdf]

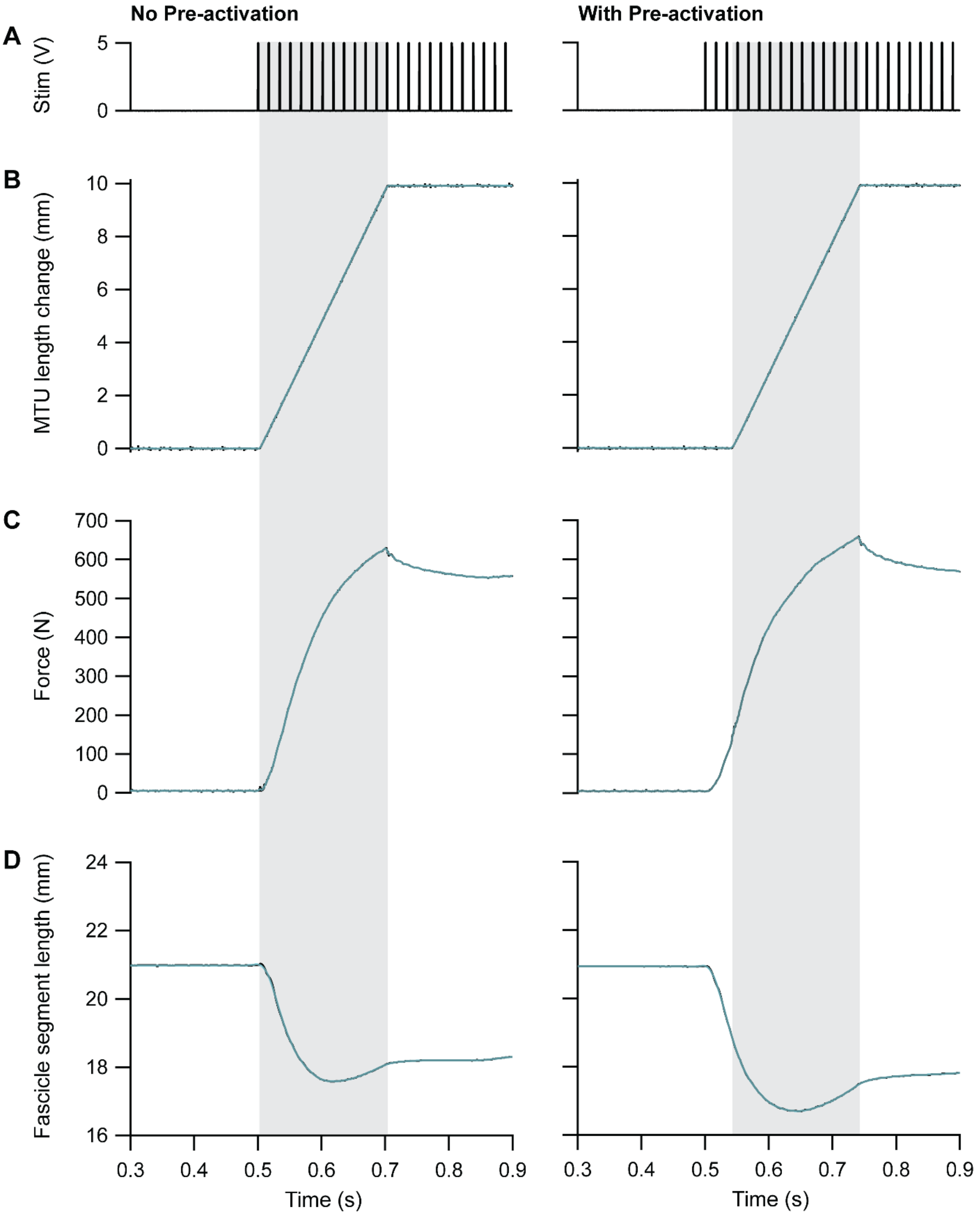

Fig. S1. Smoothing spline interpolation filter example (*n*=1). Raw data in black and filtered data in opaque blue. (A) Stimulation was recorded as an analog signal (0-5 V) from the GRASS stimulator output. A sampling rate of 10,000 Hz was set to collect data

Journal of Experimental Biology • Supplementary information

for (A) stimulation, (B) motor position, and (C) load cell signals using an NI DAQ system and IgorPro software. (D) Sonomicrometry data was collected at 508 Hz in SonoLabDS3 software. (B-D) A smoothing spline interpolation filter was applied to all data with a smoothing factor of 1 and a standard deviation that was calculated from a 500 ms window during resting baseline prior to stimulation onset and then adjusted by a multiplier that was determined upon visual inspection to best fit the data. The multipliers of the standard deviations used in this study ranged between (B) 0.825-2.85 for motor position, (C) 0.75 for load cell, and (D) 0.2-2.5 for sonomicrometry.

Journal of Experimental Biology • Supplementary information

Table S1. Summary of LG MTU parameters (*n*=6).

Bird ID	Muscle mass (g)	Time to peak force (ms)	Pre-activation duration (ms)	L_0 (mm)	F_0 (N)	Fascicle segment (%fascicle length)	Relative magnitude of the 10 mm MTU stretch (%L_0)
#098	44.6	326	40	20.47	480	76.92	48.86
#024	44.27	498	55	28.10	394	71.43	35.58
#040	27.73	267	30	32.07	214	55.56	31.18
#032	46	257	30	n/a	n/a	86.21	n/a
#014	22.13	434	50	23.33	180	45.05	42.86
#076	37	400	45	41.56	335	70.42	24.06
Mean	36.96	364	42	29.11	320.58	67.60	36.51
s.d.	9.99	96	10	8.26	124.79	14.90	9.71

Table S2. Resting baseline measures.

	Force (N)		Relative fascicle length (L/L_0)	
Bird ID	No pre-activation	With pre-activation	No pre-activation	With pre-activation
#098	5.24	5.13	1.33	1.33
#024	6.41	6.55	0.93	0.93
#040	5.56	4.43	1.13	1.13
#032	5.59	4.92	n/a	n/a
#014	1.03	2.98	1.08	1.09
#076	5.18	4.86	1.04	1.03
Mean	4.83	4.81	1.10	1.10
s.d.	1.91	1.15	0.15	0.15

Table S3. Conditions of the MTU at the start of the MTU stretch relative to the onset of muscle activation.

	Relative force (F/F_0)		Fascicle strain (ε_L)	
Bird ID	No pre-activation	With pre-activation	No pre-activation	With pre-activation
#098	0.02	0.32	0.00	0.14
#024	0.03	0.30	0.00	0.04
#040	0.04	0.20	0.00	0.02
#032	n/a	n/a	n/a	n/a
#014	0.02	0.39	0.00	0.03
#076	0.03	0.34	0.00	0.02
Mean	0.03	0.31	0.00	0.05
s.d.	0.01	0.07	0.00	0.05
	P<0.001*		P=0.022†*	

† Calculated using the Wilcoxon signed-rank test; all other tests are standard paired *t*-test. Asterisks indicate significant *P*-values (<0.05).

Table S4. Peak power input measures during MTU stretch.

	Fascicle peak power input (W kg^{-1})		MTU peak power input (W kg^{-1})		Peak power decoupling ratio	
Bird ID	No pre-activation	With pre-activation	No pre-activation	With pre-activation	No pre-activation	With pre-activation
#098	-161.23	-248.19	-708.71	-762.06	0.77	0.67
#024	-173.64	-381.80	-561.08	-661.54	0.69	0.42
#040	-187.11	-225.22	-441.58	-472.67	0.58	0.52
#032	-106.23	-150.32	-483.86	-515.86	0.78	0.71
#014	-226.69	-359.13	-687.14	-750.83	0.67	0.52
#076	-238.72	-302.72	-591.04	-630.98	0.60	0.52
Mean	-182.27	-277.90	-578.9	-632.32	0.68	0.56
s.d.	47.92	87.12	106.69	118.98	0.09	0.11
	P=0.015*		P=0.004*		P=0.015*	

The *P*-values were calculated from a standard paired *t*-test. Asterisks indicate significant *P*-values (<0.05).

Journal of Experimental Biology • Supplementary information

Table S5. Fascicle transition point (maximum fascicle shortening) during MTU stretch.

	Time to max shortening (ms) relative to start of MTU stretch		Relative force (F/F_0) at maximum fascicle shortening		Relative fascicle length (L/L_0) at maximum fascicle shortening	
Bird ID	No pre-activation	With pre-activation	No pre-activation	With pre-activation	No pre-activation	With pre-activation
#098	113.5	101.5	1.04	1.12	1.12	1.06
#024	118.9	68.3	0.84	0.87	0.91	0.85
#040	104.9	76	0.75	0.74	1.09	1.06
#032	124.9	88.4	n/a	n/a	n/a	n/a
#014	123.5	87.7	1.31	1.25	1.02	1.01
#076	75.1	32.6	0.73	0.68	1.03	1.00
Mean	110.1	75.8	0.93	0.93	1.03	1.00
s.d.	18.7	24.0	0.25	0.24	0.08	0.08
	P=0.001*		*P*=0.975		*P*=0.023*	

The *P*-values were calculated from a standard paired *t*-test. Asterisks indicate significant *P*-values (<0.05).

Table S6. Fascicle strains for phases 1 and 2.

	Phase 1 fascicle shortening strain (ε_L)		Phase 2 fascicle lengthening strain (ε_L)	
Bird ID	No pre-activation	With pre-activation	No pre-activation	With pre-activation
#098	0.22	0.27	-0.03	-0.05
#024	0.01	0.07	-0.03	-0.09
#040	0.03	0.06	-0.04	-0.06
#032	n/a	n/a	n/a	n/a
#014	0.07	0.08	-0.03	-0.08
#076	0.02	0.04	-0.04	-0.07
Mean	0.07	0.10	-0.04	-0.07
s.d.	0.08	0.09	0.01	0.02
	P=0.043†*		*P*=0.011*	

† Calculated using the Wilcoxon signed-rank test; all other tests are standard paired *t*-test. Asterisks indicate significant *P*-values (<0.05).

Journal of Experimental Biology • Supplementary information

Table S7. Measures extracted at the end of MTU stretch.

	Relative force (F/F_0)		Relative fascicle length (L/L_0)		Net fascicle strain (ε_L)	
Bird ID	No pre-activation	With pre-activation	No pre-activation	With pre-activation	No pre-activation	With pre-activation
#098	1.29	1.35	1.15	1.11	0.19	0.22
#024	1.24	1.48	0.94	0.95	-0.02	-0.02
#040	1.11	1.20	1.13	1.12	-0.01	0.00
#032	n/a	n/a	n/a	n/a	n/a	n/a
#014	1.65	1.81	1.05	1.09	0.03	0.00
#076	1.27	1.35	1.07	1.07	-0.02	-0.04
Mean	1.31	1.44	1.07	1.07	0.03	0.03
s.d.	0.20	0.23	0.08	0.07	0.09	0.10
	P=0.023*		*P*=0.897		*P*=0.893†	

† Calculated using the Wilcoxon signed-rank test; all other tests are standard paired *t*-test. Asterisks indicate significant *P*-values (<0.05).
